# Clinical Significance of Hemostatic Parameters in the Prediction for Type 2 Diabetes Mellitus and Diabetic Nephropathy

**DOI:** 10.1155/2018/5214376

**Published:** 2018-02-04

**Authors:** Lianlian Pan, Yali Ye, Mingyi Wo, Danni Bao, Fengjiao Zhu, Maoliang Cheng, Xiuwen Ni, Xianming Fei

**Affiliations:** ^1^Department of Laboratory Medicine, Sanmen Hospital of Traditional Chinese Medicine, Taizhou, Zhejiang Province, China; ^2^Department of Laboratory Medicine, Sanmen People's Hospital, Taizhou, Zhejiang Province, China; ^3^Center of Laboratory Medicine, Zhejiang Provincial People's Hospital and People's Hospital of Hangzhou Medical College, Hangzhou, Zhejiang Province, China; ^4^Department of Laboratory Medicine, Sir Run Run Hospital, Nanjing Medical University, Nanjing, Jiangsu Province, China; ^5^Department of Laboratory Medicine, Jiaxing Blood Center, Jiaxing, Zhejiang Province, China

## Abstract

It would be important to predict type 2 diabetes mellitus (T2DM) and diabetic nephropathy (DN). This study was aimed at evaluating the predicting significance of hemostatic parameters for T2DM and DN. Plasma coagulation and hematologic parameters before treatment were measured in 297 T2DM patients. The risk factors and their predicting power were evaluated. T2DM patients without complications exhibited significantly different activated partial thromboplastin time (aPTT), platelet (PLT), and D-dimer (D-D) levels compared with controls (*P* < 0.01). Fibrinogen (FIB), PLT, and D-D increased in DN patients compared with those without complications (*P* < 0.001). Both aPTT and PLT were the independent risk factors for T2DM (OR: 1.320 and 1.211, *P* < 0.01, resp.), and FIB and PLT were the independent risk factors for DN (OR: 1.611 and 1.194, *P* < 0.01, resp.). The area under ROC curve (AUC) of aPTT and PLT was 0.592 and 0.647, respectively, with low sensitivity in predicting T2DM. AUC of FIB was 0.874 with high sensitivity (85%) and specificity (76%) for DN, and that of PLT was 0.564, with sensitivity (60%) and specificity (89%) based on the cutoff values of 3.15 g/L and 245 × 10^9^/L, respectively. This study suggests that hemostatic parameters have a low predicting value for T2DM, whereas fibrinogen is a powerful predictor for DN.

## 1. Introduction

The development of T2DM is a chronic process with approximately a decade-long latent period before the clinical onset of the disease [1]. As the third leading cause of mortality, diabetes seriously threatens the human health worldwide, and there are many serious complications of diabetes such as microvascular complications [2]. In these complications, diabetic nephropathy is the main one which could lead to kidney failure [3]. Diabetic kidney disease is one of the critical problems of diabetes mellitus in which the prevalence has been increasing worldwide [4]. Diabetic nephropathy is a unique predictor of mortality in both insulin-dependent and insulin-independent diabetes [5]. Therefore, early identification of the diabetes and microvascular complication risk provides an opportunity to introduce preventive interventions to stop or delay disease onset [6], which would be more important to decrease the morbidity and mortality of T2DM patients with microvascular complications.

Many biological markers or biomarkers, such as C-reactive protein, gamma-glutamyl transpeptidase, or adiponectin, are associated with the risk of developing T2DM [7]. Moreover, some indicators such as high-sensitive C-reactive protein, C-peptide, or total bilirubin can reflect the presence of microvascular damage in T2DM patients [8] and are associated with the risk of microangiopathy [9, 10]. It has been recognized that many biomarkers play potential roles in diagnosis and prognosis of diabetic nephropathy [11]. Moreover, patients with diabetes could show hypercoagulability and high levels of coagulation factors [12], and diabetes mellitus, including type 1 and type 2, is associated with the hypercoagulable state through increased thrombotic tendencies [13], even exhibiting prothrombotic milieu which is due to upregulation of coagulation factors and prolongation of clot lysis [14]. Increased concentration of coagulation factors is widely reported in type 2 diabetes mellitus [15]. D-dimer and other coagulating parameters are also observed in patients with diabetes mellitus [16, 17]. Although many studies mentioned above have revealed the association of hemostatic and coagulation abnormalities with T2DM, there were few studies focusing on the relationship between hemostatic parameters and risk of diabetic nephropathy in T2DM. Therefore, the aim of this study was to explore the clinical prediction of hemostatic parameters for T2DM and diabetic nephropathy.

## 2. Materials and Methods

### 2.1. Patient Population

A total of 297 patients with T2DM, including 189 males and 108 females aged 37–85 (mean: 57 ± 21) years from the Departments of Endocrinology, Sanmen Hospital of Traditional Chinese Medicine, Sanmen People's Hospital of Taizhou City, Zhejiang Province, and Zhejiang Provincial People's Hospital, China, between October 2015 and December 2016, were finally enrolled in this retrospective study. All patients were Han Chinese and diagnosed according to the criteria in diabetes guideline 2013 of the China Diabetes Association [18]. The baseline evaluation before treatment included clinical assessment (clinical history, risk factors, data of physical examination, and clinical and biological data during hospitalization) and laboratory tests. The inclusion criteria included (1) diabetes without complications and (2) diabetes with nephropathy. The exclusion criteria included (1) other diabetic complications, (2) primary liver and kidney dysfunctions, (3) postoperation, (4) hypertension, (5) cardiovascular and cerebrovascular diseases, (6) malignancies, (7) inflammation and infections, (8) thrombotic diseases, and (9) drug therapy influencing the hemostatic parameters (antithrombotic agents: warfarin, heparin, aspirin, some traditional Chinese medicines, etc., and hemostatic agents: hemocoagulase, vitamin K_1_, etc.) in two weeks before sample collection. T2DM patients were divided into two groups including T2DM without complications group (202 patients) and T2DM with nephropathy group (95 patients). For comparison, 141 healthy controls matched for age, gender, and race were included in the study. The controls came from the healthy management center of the three hospitals and also did not take any abovementioned antithrombotic and hemostatic agents in two weeks before sample collection. Informed consent was obtained from the controls and patients or their relatives, and the study was approved by the institutional review board of the three hospitals.

### 2.2. Laboratory Assay

Samples were collected and prepared as described previously [19]. In brief, venous blood of patients was collected in the morning after an overnight fast for measurements of routine hemostatic parameters including prothrombin time (PT), international normalized ratio (INR), activated partial thromboplastin time (aPTT), thrombin time (TT), fibrinogen (FIB), D-dimer (D-D), platelet (PLT) count, and fibrinogen-D-dimer ratio (FDR) before treatment, respectively. For coagulation analysis, blood sample (9 vol) was collected into vacutainer tubes (Becton Dickinson, Mountain View, CA, USA) containing 0.129 mol/L trisodium citrate (1 vol) and mixed completely. Platelet-poor plasma was obtained by centrifugation at 1500*g* at room temperature for 15 minutes. Subsequently, the measurements of PT, aPTT, TT, FIB, and D-D were performed with a coagulation analyzer (CS-5100, Sysmex, Japan) and the commercial reagents (Siemens Healthcare Diagnostics Products GmbH) within two hours after sample collection, respectively; INR and FDR were also calculated. For PLT analysis, two milliliters of blood sample was collected into vacutainer tubes (Becton Dickinson, Mountain View, CA, USA) containing 3.6 mg EDTA-K2 and mixed completely. Platelet count was performed with a blood cell analyzer (BC-6800, Mindray, China) in one hour after sample collection. Routine biochemical parameters (including cystatin C, thyroid-stimulating hormone, fasting serum glucose, C-peptide, glycosylated hemoglobin, serum high-density lipoprotein, total cholesterol, and albumin) were measured. Estimated glomerular filtration rate (eGFR) was calculated according to the formula eGFR = 133 × (CysC/0.8)^−0.449(−1.328 if CysC > 0.8mg/L)^ × 0.996^age^ × (0.932 if female) [20]. And the biological and clinical data of all patients (age and gender, diabetic complications, and T2DM-related and pathological data) before treatment were collected and reviewed.

### 2.3. Statistical Analysis

One-way analysis of variance (ANOVA) was used to analyze the difference of the data among control, T2DM, and DN groups. For samples of normal and nonnormal distribution data (PLT and D-D), Student's *t*-test and Mann–Whitney *U* test were used, respectively. And the chi-square test was used for categorical variables. Univariate and multivariate logistic regression analyses were performed to calculate the odds ratio and 95% confidence interval of all hemostatic parameters to screen the risk factors for T2DM and DN. Receiver operating characteristic curve was constructed, and the area under curves was calculated to demonstrate the predicting power of the independent risk factors. Statistical analyses were performed using statistical package SPSS 20.0 (SPSS, Chicago, Illinois, USA). *P* value of less than 0.05 was considered statistically significant.

## 3. Results

### 3.1. Basic Clinical Characteristics of Patients

Basic clinical characteristics of the controls and patients are listed in Table 1. Compared with controls and patients with T2DM without complications, eGFR, THS, C-peptide, and HDL levels in patients with DN were significantly increased (*P* < 0.05). RDW, HbA1c, and CHOL levels in DN patients were higher than those in controls (*P* < 0.01), and there was no statistical difference between the two T2DM groups (*P* > 0.05).

### 3.2. Comparisons of Hemostatic Parameters between Patients with T2DM and Healthy Controls

Patients without complications exhibited significantly shortened activated partial thromboplastin time (aPTT), decreased platelet (PLT) count, and increased D-dimer (D-D) level compared with controls (*P* < 0.01). DN patients demonstrated increased fibrinogen (FIB), PLT, and D-D levels compared with patients without complications (*P* < 0.001 for all variables). Other parameters did not exhibit significant difference between the groups. The detailed data are presented in Table 2.

### 3.3. Results of Regression Analysis of Hemostatic Parameters for T2DM and Diabetic Nephropathy

The association of each parameter with the risk for T2DM and diabetic nephropathy was analyzed separately. Univariate analysis showed that INR, aPTT, D-D, DFR, and PLT among the laboratory variables of hemostatic assays were the risk factors for T2DM without complications (*P* < 0.01 for all variables, but *P* < 0.05 for D-D). However, multivariate analysis revealed that both aPTT and PLT were independently correlated with T2DM without complications (aPTT (OR: 1.320, 95% CI: 1.137–1.650) and PLT (OR: 1.211, 95% CI: 1.021–1.387), *P* < 0.01, resp.). Data are presented in Table 3. Further univariate and multivariate analyses were performed for all patients including patients without complications and with nephropathy. The results demonstrated that FIB and PLT were the independent risk factors for T2DM with nephropathy (OR: 1.611, 95% CI: 1.338–2.122 and OR: 1.194, 95% CI: 1.039–1.411; *P* < 0.01, resp.). Detailed results are presented in Table 4.

### 3.4. Results of ROC Curve Analysis of the Independent Risk Factors for the Prediction of T2DM and Diabetic Nephropathy

ROC curve was used to evaluate the predicting power of the independent risk factors for T2DM and diabetic nephropathy. For T2DM without complications, the area under ROC curve (AUC) of aPTT and PLT demonstrated that there was low total predicting power (aPTT: 0.592, 95% CI: 0.517–0.667 and PLT: 0.647, 95% CI: 0.577–0.718), and the sensitivity was also low (60% and 65%, resp.), whereas there was high specificity (89% and 86%, resp.) according to the cutoff values (24.3 seconds and 176 × 10^9^/L, resp.). Based on the cutoff values, combinations of aPTT and PLT could increase the predicting power (AUC: 0.694, 95% CI: 0.634–0.755). Further study exhibited the high predicting power (AUC: 0.874, 95% CI: 0.830–0.921), high sensitivity (85%), and high specificity (76%) of FIB for nephropathy based on the cutoff value (3.15 g/L). However, PLT showed low predicting power (AUC: 0.564, 95% CI: 0.477–0.602) and sensitivity (60%) but high specificity (89%) for nephropathy according to the cutoff value (205 × 10^9^/L). When combining FIB and PLT, the predicting power increased (AUC: 0.883, 95% CI: 0.840–0.933; sensitivity: 90%; and specificity: 72%). Data are presented in Table 5.

## 4. Discussion

Patients with T2DM or DN may exhibit the abnormalities of biochemical parameters as what the results demonstrated in the present study, which were sure to be associated with the metabolic disorders and consistent with those of the studies reported by other authors [3]. It has been proved that various biomarkers, including hematology, biochemistry, immunology, and hemostasis, would be influenced in the development of diabetes mellitus (DM). Therefore, some parameters might be valuable to evaluate the progression of DM. Enhanced activation of the clotting cascade has recently been implicated as an important contributing factor for the occurrence of vascular complications in DM, and DM is associated with disturbances in hemostasis [21]. In this study of risk and prediction for T2DM patients with or without nephropathy, the results demonstrated that there were decreased aPTT and PLT and increased D-D levels in T2DM patients without complications compared with controls, which indicated that diabetics had decreased level of PLT count and relatively shortened aPTT as well as elevated D-D level. And other reports seem to partly prove the present results [22, 23]. At the same time, increased FIB, PLT, and D-D as well as decreased FDR levels were found in patients with DN compared with those without complications. The results demonstrated that hemostatic abnormalities were associated with T2DM development and disease progression to some extent. Overall, these results implied a general tendency towards a higher degree of hypercoagulability in T2DM patients and patients with DN. Therefore, the present study seemed to reveal that some hemostatic parameters such as aPTT, FIB, PLT, and D-D might be useful as the risk and predicting indicators for T2DM and DN, which was in accordance with other reports [24, 25]. Therefore, it was necessary to further explore whether they were actually valuable predictors for T2DM and DN.

Various studies have demonstrated that T2DM and diabetic microvascular disease patients, especially those with diabetic nephropathy, would exhibit a hypercoagulable state and hemostatic abnormalities [21, 26, 27]. Therefore, further study was performed to evaluate the significance of hemostatic parameters in predicting T2DM and DN. Multivariate regression analysis showed that only aPTT and PLT had higher OR values in predicting T2DM than other hemostatic parameters, respectively. At the same time, only FIB and PLT exhibited higher OR values in the prediction for DN. The results indicated that shortened aPTT and decreased PLT count were the risk factors of T2DM without complications and increased FIB and PLT had the predicting values for DN. Therefore, the study also further revealed that routine hemostatic assays might be valuable for monitoring the occurrence of T2DM and DN. To further assess the predicting power of the three risk factors mentioned above, ROC curve analysis was used. From the ROC curve analysis, although aPTT and PLT showed a higher specificity of 89% and 86%, respectively, the area under ROC curve (AUC) of 0.592 and 0.647, respectively, demonstrated that they were not valuable parameters in the prediction for T2DM without complications because of the low predicting power. Although there was significant difference for APTT and PLT between diabetic and nondiabetic patients, their practical significance in the prediction for diabetes mellitus was lower. However, FIB and PLT exhibited the AUC of 0.874 and 0.564, respectively, in the prediction for nephropathy in T2DM patients. And based on the cutoff value of 3.15 g/L, FIB exhibited high sensitivity and specificity of 85% and 76%, respectively, in the prediction for DN, whereas PLT has only a high specificity of 89% with a low sensitivity of 60%. The results revealed that FIB had higher predicting power than PLT for DN in T2DM patients. Le et al. also revealed the similar results in their study on young T2DM patients [21]. Therefore, T2DM patients with high FIB level may be at risk for DN, which indicates that it is useful for FIB to predict nephropathy in T2DM patients.

Clinical practice has proved that combinations of several diagnostic parameters could increase the diagnostic and predicting power. As the present study revealed, the predicting significance of aPTT and PLT was low for T2DM, whereas FIB had high predicting significance for DN. Therefore, it would be important to investigate whether the combination of FIB and PLT had higher predictive power for DN in T2DM patients than their single use. As expected, the results demonstrated a higher AUC of 0.883, a sensitivity of 90%, and a specificity of 72% for predicting DN when using their cutoff values, respectively, than those of FIB or PLT single use. From the results, the study further indicated that combining FIB and PLT measurements may serve as a more useful tool in the prediction for nephropathy in T2DM patients.

There are at least two limitations concerning the present study. Firstly, there probably were some nephropathy patients with other complications and T2DM patients with complications which were not diagnosed before treatment. Therefore, the laboratory data from those patients would cause some uncertain results and might potentially increase the predicting power of the hemostatic parameters. Secondly, the percentage of patients with serious nephropathy also might potentially increase the predicting power, and patients were not stratified based on disease condition of nephropathy in this study. Therefore, it was unclear whether there was significant difference for levels of hemostatic parameters between slight and serious DN. Although there were limitations, the present study also revealed the valuable predicting significance of fibrinogen and the combination of fibrinogen and platelet count in the prediction for nephropathy in T2DM patients.

## 5. Conclusion

This study suggests that hemostatic parameters have a lower predicting value for type 2 diabetes mellitus, and fibrinogen is the independent risk factor with high predicting power for nephropathy in patients with type 2 diabetes mellitus, and a combination of fibrinogen and platelet count can increase the predicting power. However, further multicentric and controlled prospective studies on large groups of patients may give more definite results.

## Figures and Tables

**Table 1:** Clinical characteristics of controls and patients with type 2 diabetes mellitus.

	Controls	T2DM without complications	Diabetic nephropathy	*P* value
*n*	141	202	95	
Systolic pressure (mmHg)	106 ± 10	107 ± 12	105 ± 12	>0.05
eGFR (mL/min)	75.3 ± 10.4	79.2 ± 13.2	95.6 ± 20.1^∗^	<0.01
WBC (g/L)	4.67 ± 1.01	5.12 ± 1.20	5.04 ± 1.30	>0.05
HGB (g/L)	120 ± 12	121 ± 13	118 ± 14	>0.05
RDW (%)	12.3 ± 1.1	12.9 ± 1.4	13.2 ± 1.5^▲^	<0.01
TSH (*μ*mmol/L)	1.22 ± 0.51	1.44 ± 0.46	1.55 ± 0.49^∗^	<0.05
C-peptide (nmol/L)	0.61 ± 0.22	1.02 ± .031	1.21 ± 0.47^∗^	<0.01
HbA1c (%)	3.02 ± 1.33	6.22 ± 2.65	6.33 ± 2.56^▲^	<0.01
HDL (mmol/L)	1.01 ± 0.33	0.89 ± 0.35	0.77 ± 0.30^∗^	<0.01
CHOL (mmol/L)	2.35 ± 1.44	3.01 ± 1.69	3.43 ± 1.77^▲^	<0.01
ALB (g/L)	45.3 ± 12.1	42.4 ± 10.5	40.0 ± 11.2	<0.05

Data were presented as mean and standard deviation (SD) or median (range). T2DM: type 2 diabetes mellitus; eGFR: estimated glomerular filtration rate; WBC: white blood cell; HGB: hemoglobin; RDW: red cell distribution width; TSH: thyroid-stimulating hormone; HbA1c: glycosylated hemoglobin A1c; HDL: high-density lipoprotein; CHOL: cholesterol; ALB: albumin. *P* value: comparisons of the three groups by one-way analysis of variance. ^∗^*P* < 0.05 compared with controls and patients with T2DM without complications; ^▲^*P* < 0.05 compared with controls.

**Table 2:** Comparisons of hemostatic parameters of T2DM patients with those of healthy controls.

Variables	Controls	Without complications	Diabetic nephropathy	*P* value
*n*	141	202	95	
Gender (M/F)	78/53	121/81	44/30	>0.05
Age (median) (years)	56 (33–79)	57 (31–82)	67 (35–87)	>0.05
PT (s)	11.02 ± 0.56	11.4 ± 2.12	12.2 ± 4.62	>0.05
INR	0.98 ± 0.05	1.02 ± 0.19	1.06 ± 0.45	>0.05
APTT (s)	27.5 ± 2.56	25.1 ± 3.7^▲^	24.7 ± 4.8	>0.05
FIB (g/L)	2.65 ± 0.40	2.75 ± 0.83	3.51 ± 1.33^∗^	<0.01
PLT (10^9^/L)	211 (89–323)	183 (43–447)^▲^	207 (79–709)^∗^	<0.01
D-D (median) (mg/L)	0.14 (0.03–0.54)	0.18 (0.04–4.27)^▲^	0.48 (0.06–10.31)^∗^	<0.01
FDR (median) (g/mg)	18.94 ± 3.11	17.19 ± 4.12	7.31 ± 2.12^∗^	<0.01
TT (s)	17.6 ± 0.83	17.6 ± 0.95	17.5 ± 1.21	>0.05

Data were presented as mean and standard deviation (SD) or median (range). T2DM: type 2 diabetes mellitus; PT: prothrombin time; INR: international normalized ratio; aPTT: activated partial thromboplastin time; FIB: fibrinogen; PLT: platelet count; D-D: D-dimer; FDR: fibrinogen-D-dimer ratio; TT: thrombin time. *P* value: comparisons of the three groups by one-way analysis of variance. ^∗^*P* < 0.01 compared with controls and patients with T2DM without complications; ^▲^*P* < 0.05 compared with controls.

**Table 3:** Logistic regression analysis of hemostatic assays in T2DM without complications.

Variables	Univariate regression			Multivariate regression		
	OR	95% CI	*P*	OR	95% CI	*P*
PT	1.762	(1.168–2.659)	0.007	0.958	(0.915–2.128)	0.145
INR	3.521	(2.614–4.195)	0.000	0.950	(0.911–2.015)	0.105
APTT	1.555	(1.285–1.730)	0.000	1.320	(1.137–1.650)	0.002
FIB	1.338	(0.886–2.021)	0.167	0.872	(0.422–1.831)	0.712
PLT	1.388	(1.188–1.597)	0.000	1.211	(1.021–1.387)	0.003
D-D	1.002	(1.000-1.004)	0.019	0.999	(0.993–1.006)	0.887
FDR	1.006	(1.001–1.011)	0.002	0.996	(0.978–1.015)	0.708
TT	0.945	(0.768–1.260)	0.698	1.119	(0.764–1.638)	0.563

T2DM: type 2 diabetes mellitus; PT: prothrombin time; INR: international normalized ratio; aPTT: activated partial thromboplastin time; FIB: fibrinogen; PLT: platelet count; D-D: D-dimer; FDR: fibrinogen-D-dimer ratio; TT: thrombin time; OR: odds ratio; CI: confidence interval.

**Table 4:** Regression analysis of hemostatic assays in T2DM patients with nephropathy.

Variables	Univariate regressions			Multivariate regression		
	OR	95% CI	*P*	OR	95% CI	*P*
PT	1.080	(0.975–1.197)	0.139	0.982	(0.802–4.804)	0.236
INR	1.4981	(0.604–3.715)	0.383	1.101	(0.588–2.566)	0.412
APTT	1.037	(0.970–1.110)	0.925	0.981	(0.848–3.778)	0.931
FIB	1.972	(1.437–2.669)	0.000	1.611	(1.338–2.122)	0.001
PLT	1.305	(1.101–1.509)	0.000	1.194	(1.039–1.411)	0.009
D-D	1.122	(1.008–1.212)	0.000	1.055	(0.939–1.124)	0.312
FDR	1.001	(1.000–1.002)	0.007	0.995	(0.974–1.036)	0.479
TT	0.933	(0.704–1.237)	0.631	0.958	(0.681–1.377)	0.805

T2DM: type 2 diabetes mellitus; PT: prothrombin time; INR: international normalized ratio; aPTT: activated partial thromboplastin time; FIB: fibrinogen; PLT: platelet count; D-D: D-dimer; FDR: fibrinogen-D-dimer ratio; TT: thrombin time; OR: odds ratio; CI: confidence interval.

**Table 5:** Predicting power of the independent risk factors for T2DM and DN.

Complications	Variables	AUC	*P*	95% CI		Cutoff value	Sensitivity (%)	Specificity (%)
				Lower	Upper			
Noncomplications	APTT	0.592	0.018	0.517	0.667	24.3 s	60	89
	PLT	0.647	0.000	0.577	0.718	176 × 10^9^/L	65	86
	aPTT and PLT	0.694	0.00	0.634	0.755	ND	70	82

Nephropathy	FIB	0.874	0.000	0.830	0.921	3.15 g/L	85	76
	PLT	0.564	0.133	0.477	0.602	205 × 10^9^/L	60	89
	FIB and PLT	0.883	0.000	0.840	0.933	ND	90	72

AUC: area under curve; T2DM: type 2 diabetes mellitus; DN: diabetic nephropathy; CI: confidence interval; aPTT: activated partial thromboplastin time; PLT: platelet count; FIB: fibrinogen; ND: no data; CI: confidence interval.
